# Reduced social attention in autism is magnified by perceptual load in naturalistic environments

**DOI:** 10.1002/aur.2829

**Published:** 2022-10-07

**Authors:** Amanda J. Haskins, Jeff Mentch, Thomas L. Botch, Brenda D. Garcia, Alexandra L. Burrows, Caroline E. Robertson

**Affiliations:** ^1^ Department of Psychological & Brain Sciences Dartmouth College Hanover New Hampshire USA; ^2^ Speech and Hearing Bioscience and Technology Harvard University Boston Massachusetts USA; ^3^ McGovern Institute for Brain Research, MIT Cambridge Massachusetts USA

**Keywords:** adults, attention, eye movement, sensory, social cognition

## Abstract

Individuals with autism spectrum conditions (ASC) describe differences in both social cognition and sensory processing, but little is known about the causal relationship between these disparate functional domains. In the present study, we sought to understand how a core characteristic of autism—reduced social attention—is impacted by the complex multisensory signals present in real‐world environments. We tested the hypothesis that reductions in social attention associated with autism would be magnified by increasing perceptual load (e.g., motion, multisensory cues). Adult participants (*N* = 40; 19 ASC) explored a diverse set of 360° real‐world scenes in a naturalistic, active viewing paradigm (immersive virtual reality + eyetracking). Across three conditions, we systematically varied perceptual load while holding the social and semantic information present in each scene constant. We demonstrate that reduced social attention is not a static signature of the autistic phenotype. Rather, group differences in social attention emerged with increasing perceptual load in naturalistic environments, and the susceptibility of social attention to perceptual load predicted continuous measures of autistic traits across groups. Crucially, this pattern was specific to the social domain: we did not observe differential impacts of perceptual load on attention directed toward nonsocial semantic (i.e., object, place) information or low‐level fixation behavior (i.e., overall fixation frequency or duration). This study provides a direct link between social and sensory processing in autism. Moreover, reduced social attention may be an inaccurate characterization of autism. Instead, our results suggest that social attention in autism is better explained by “social vulnerability,” particularly to the perceptual load of real‐world environments.

## Lay Summary

Real‐world sensory contexts place incredible demands on selective attention. Autistic individuals report a particular challenge navigating contexts with high perceptual load, but little is known about its impact on classic, social signatures of autism. Here, we used eyetracking in immersive virtual reality to test the impact of perceptual load on social attention in naturalistic contexts. Overall, we found that group differences in social attention emerge with increasing real‐world perceptual load, revealing a novel link between social and sensory processing in autism.

## INTRODUCTION

Research has demonstrated that differences in sensory processing are near‐universal among individuals with autism, suggesting that both social and sensory traits are core characteristics of the condition (Robertson & Baron‐Cohen, 2017). Yet, beyond self‐report evidence in population studies that social and sensory traits are correlated (Horder et al., 2014; Tavassoli et al., 2014), little is known about their causal connection. This is particularly the case in complex, real‐world environments, where sensory and social processing demands co‐occur in daily life. Do sensory and social processing demands interact in real‐world environments? If so, does this interaction differentially impact individuals across the autism spectrum?

Many hallmark signatures of atypical social behavior in autism—reduced eye contact, difficulty interpreting gestures, and vocal prosodic cues (American Psychiatric Association, 2013)—are foundationally linked to social attention (Haith et al., 1977). Differences in social attention have been studied extensively in autism using computer‐based eyetracking paradigms (Frazier et al., 2017). Gaze behavior is a reliable (de Haas et al., 2019), even heritable (Kennedy et al., 2017) signal that can be measured passively and early in development (Gredebäck et al., 2010). As such, eyetracking has been viewed as a potential diagnostic instrument in the autism field (Frazier et al., 2021; Murias et al., 2018), capable of capturing subtle differences in social attention that foreshadow future behavioral challenges (e.g., difficulty initiating and maintaining social interaction) (Jones et al., 2008; Klin et al., 2002). Perplexingly, however, group differences in social attention observed in these lab‐based settings are often small (Chita‐Tegmark, 2016) and inconsistent (Guillon et al., 2014) compared to clinical reports, failing to reflect the magnitude of difficulty that individuals experience in everyday social contexts. Why is such a well‐known real‐world signature of autism—reduced social attention—so challenging to consistently detect in empirical studies?

One reason for the discrepancy between real‐world and lab‐based observations may be that social and sensory traits are linked in autism, and thus, autistic differences in social attention do not fully or consistently manifest in the absence of real‐world perceptual load. Relative to everyday life, the perceptual processing demands in traditional eyetracking paradigms are low: small, simple stimuli (e.g., decontextualized faces or objects) are presented briefly on a computer screen to a passive viewer (e.g., Wang et al., 2015). In contrast, in real‐world environments, attention occurs in a complex informational landscape, and in service of real‐world behavior (Hayhoe, 2017). Successful social behavior relies on selectively attending to socially relevant cues in the presence of competing information: the dynamic, multisensory perceptual features of the environment in which theses cues are embedded. Because these perceptual features are often excluded from traditional eyetracking paradigms, little is known about social attention in complex, multisensory environments where autistic traits are most apparent in daily life.

Previous studies have shown that perceptual load impacts selective attention in well studied visual tasks, such as visual search and inattentional blindness (Lavie et al., 2014). For example, in visual search (e.g., find a target letter in a search array), neurotypical individuals are more susceptible to distractor interference (e.g., presentation of a non‐target letter outside of the array) under low perceptual load (e.g., smaller array size) than they are under high perceptual load (e.g., larger array size), suggesting that task‐irrelevant distractors are no longer processed when the perceptual demands in a visual task exceed an individual's perceptual capacity (Lavie, 1995). Interestingly, autistic participants are differentially impacted by perceptual load in such tasks: distractors are still processed to a greater degree in high load conditions, compared with controls (Remington et al., 2009; Swettenham et al., 2014), even across sensory modalities (Tillmann & Swettenham, 2017). These results have been taken as evidence for enhanced perceptual capacity in autism and linked to the real‐world experience of sensory *over*load (Remington & Fairnie, 2017). In other words, although enhanced perceptual abilities may confer real‐world benefits beyond lab‐based tasks, intuitively, a less filtered perceptual experience may negatively impact an individual. Indeed, autistic adults report that their unique perceptual experiences cause cascading physical, emotional, and cognitive effects in daily life (MacLennan et al., 2021). Yet, the impact of perceptual load in real‐world environments on selective attention, and more specifically social attention has not been systematically investigated.

Here, we sought to narrow the gap between real‐world environments and lab‐based eyetracking paradigms with a novel combination of immersive virtual reality (VR), eyetracking, and computational modeling to study the impact of perceptual load on social attention in autism. Specifically, we tested the hypothesis that group differences in social attention would be magnified by increasing perceptual load in real‐world environments, and that the susceptibility of social attention to perceptual load would predict continuous measures of autistic traits. Participants viewed immersive, real‐world scenes in three experimental conditions that systematically increased perceptual load, while visual semantic information was held constant. In brief, we find that group differences are indeed magnified in increasingly complex viewing conditions, and the degree of this interaction scales with autistic traits across groups. These results have an important implication for understanding the relationship between social and sensory traits in autism, suggesting the need to reconceptualize divergent social attention in autism as vulnerable to perceptual load, rather than globally reduced.

## METHODS

### Participants

Forty participants (19 autism spectrum conditions [ASC], 21 typically developed [TD]; Table 1) were recruited to participate in this experiment. Participants were recruited from the local community and/or referred for participation from local health providers. Participants were recruited based on the following: (1) normal or corrected vision, and (2) no history of seizures in the last 5 years. Additionally, ASC participants were recruited based on having an established ASC diagnosis, confirmed by research‐reliable administration of the Autism Diagnostic Observation Schedule (ADOS‐2), Module 4 (Lord et al., 2000). This study was performed in accordance with relevant guidelines and regulations, all experimental procedures were approved by the Dartmouth College Committee for the Protection of Human Subjects (CPHS) and Massachusetts Institute of Technology Committee on the Use of Humans as Experimental Subjects (COUHES), and all participants gave informed consent to participate.

**TABLE 1:** Psychometric data

		Age	NVIQ	AQ	ADOS‐2
ASC	*N* = 19 (3 female)				
	Minimum	17.00	82.00	16.00	7.00
	Maximum	44.00	132.00	45.00	17.00
	Mean	28.67	107.28	30.28	10.61
	SD	7.55	13.06	9.21	3.22
Controls	*N* = 21 (12 female)				
	Minimum	18.00	72.00	8.00	
	Maximum	47.00	132.00	23.00	
	Mean	23.71	114.14	17.62	
	SD	8.68	15.28	4.40	

*Note*: No significant differences were observed between the two groups in age or NVIQ (*p* > 0.05).

Abbreviations: ADOS‐2, Autism Diagnostic Observation Schedule; AQ, autism spectrum quotient; NVIQ, nonverbal intelligence.

### Materials—Psychometric assessments

All participants completed the Kaufman Brief Intelligence Test (KBIT‐2) nonverbal intelligence (NVIQ) subtest (Kaufman, 1990) and the autism spectrum quotient (AQ) self‐report questionnaire (Baron‐Cohen et al., 2001). Participants in the ASC and TD groups did not differ significantly in age (*t*(37) = 1.89, *p* = 0.07) or nonverbal intelligence (*t*(37) = 1.49, *p* = 0.14). See Table 1 for group psychometric comparisons.

### Materials—Stimuli

**FIGURE 1:**
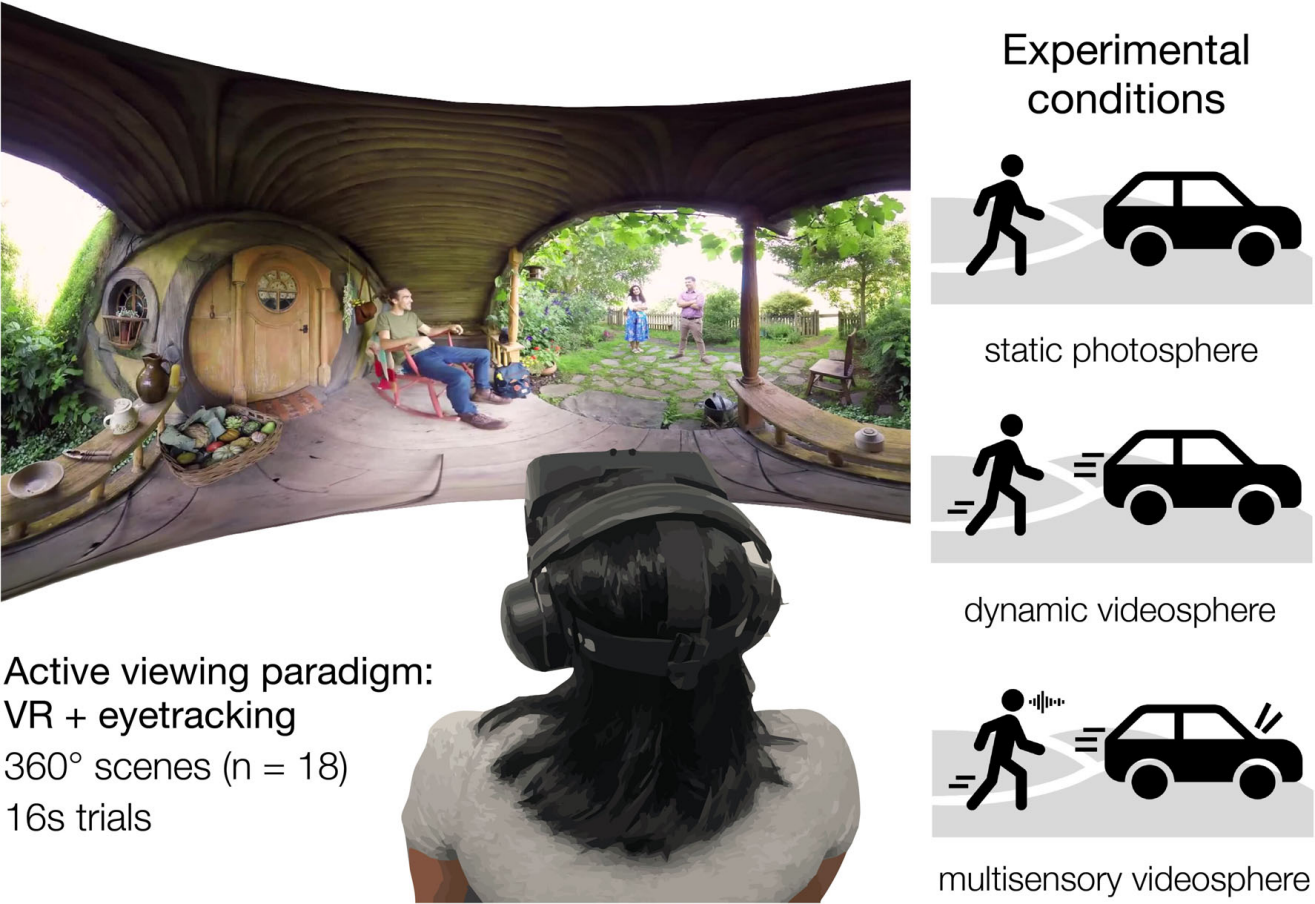
Experimental paradigm and perceptual load conditions. Using a headmounted virtual reality (VR) display with in‐headset eyetracking, participants actively explored 18 immersive, real‐world scenes in three conditions that systematically varied real‐world perceptual load. Every scene was explored three times, once in each of the three perceptual load conditions. The first condition was a static, 360° photosphere; in the second condition (dynamic videosphere) we introduced dynamic motion cues; and in the final condition (multisensory videosphere) we introduced audio information. On each trial, participants' task was simply to “look around the scene, just like you would in real life”.

Stimuli consisted of 360° panoramic “videospheres” of real‐world scenes, sourced from an online media sharing platform (www.youtube.com). Stimuli depicted a diverse set of indoor and outdoor settings (e.g., a cafe, a backyard) with content including people, objects, and scenery. To curate this stimulus set, an independent pilot experiment was conducted in which control participants (*N* = 8) viewed a large set of >150 photospheres (including single frames from videospheres). From this larger stimulus set, we identified videospheres in which both social (i.e., humans) and nonsocial entities (e.g., tools, cars, doors) were reliably fixated by pilot participants. In other words, group attention maps from these participants revealed that each scene in the final stimulus set was composed of a diverse landscape of naturalistic content, including both salient social and nonsocial content of broad interest to more than a single individual. The final stimulus set contained 18 scenes (Figure S1), which we presented to each participant in three perceptual load conditions (Figure 1): static photosphere (static frame from original source), dynamic videosphere (original source, no sound), and a multisensory audio‐videosphere (original source). To be able to parametrically vary the perceptual complexity present in a scene while holding constant the spatial distribution of visual semantic information, we only included videospheres in which social information (i.e., people) remained relatively stationary during the videos (i.e., gesturing in place, but not walking across the scene).

### Materials—Continuous models of social information in immersive scenes

**FIGURE 2:**
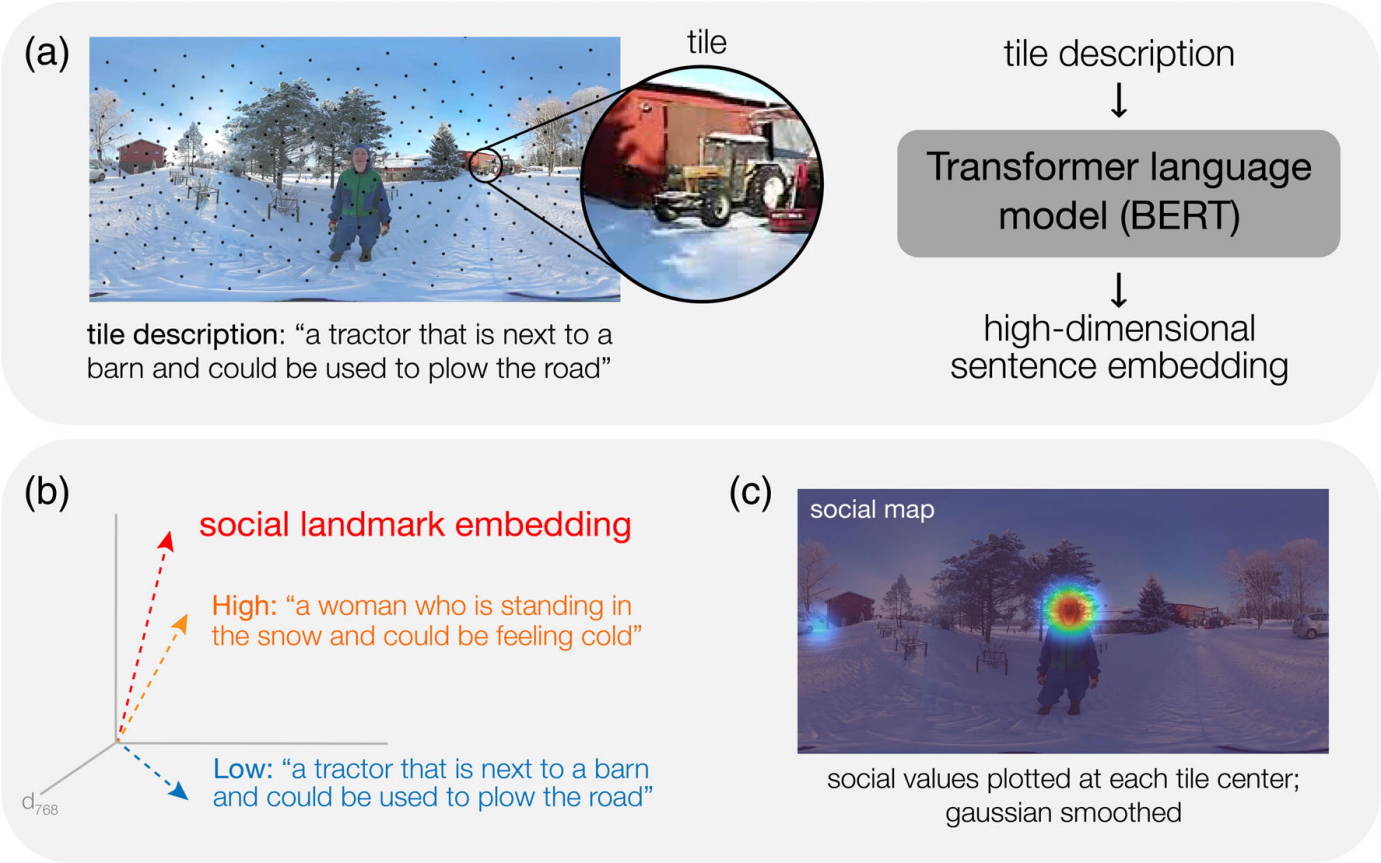
Generation of continuous, contextually informed social maps. (a) Each equirectangular scene image was decomposed into 300 image tiles. Participants on Amazon Mechanical Turk provided verbal descriptions of each tile, relating its content to the broader scene context. Tile descriptions were transformed into sentence‐level embeddings using a computational language model (bidirectional encoder representations from transformers; BERT). (b) Sentence embeddings for each tile were compared to a “social landmark” embedding in high‐dimensional semantic space. See Table 2 for example landmark sentences. This comparison yielded a similarity value which was plotted on the equirectangular image at the center of the tile for which the embedding was obtained. Here, the orange sentence is closer in semantic space to the social landmark than the blue sentence; in the social map, the tile corresponding to the orange sentence is assigned a higher value. (c) Social values were scaled and smoothed with a variable‐width Gaussian to obtain one continuous model of social information for each scene. See Figure S6 for additional example maps.

To model the continuous distribution of social information in each scene, we combined rich, contextually informed descriptions provided by online human raters (*N* = 2650) and state‐of‐the‐art computational language modeling (BERT) (Devlin et al., 2019) (Figure 2). Briefly, each scene was segmented into smaller image tiles, and online participants provided contextually informed text descriptions of the content in each tile. We then combined these descriptions with state‐of‐the‐art computational language modeling (BERT) (Devlin et al., 2019) to approximate the continuous distribution of three domains of real‐world information in each scene—social (primary analysis), object (control analysis), and place (control analysis)—via a single modeling process.

We first obtained descriptions of the semantic information in a given region of each photosphere from online participants on Amazon Mechanical Turk (*N* = 2650). Each photosphere was first decomposed into 300 tiles (Figure 2a), which evenly sampled the photosphere and accounted for photosphere distortion in two‐dimensional maps. Each tile was then labeled by five online participants. Because viewers make fixations near the poles so rarely (Sitzmann et al., 2018), labels were not obtained for the 20% of tiles located at extreme latitud. Participants saw a tile alongside an equirectangular display of the photosphere from which it was taken, enabling participants to provide contextually informed labels for each tile (Figure 2a). Underneath the tile and scene image, participants completed a fill‐in‐the‐blank task with three responses:
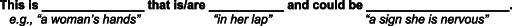


This ensured that all labels shared the same general grammatical structure, and that the predominant variation across labels was in semantic content rather than syntactic form. Participants were prevented from labeling the same tile more than once. Responses that utilized the task example language (e.g., “a crowd of people”) or words from non‐English languages were excluded from the analysis. Participants' labels were preprocessed to remove spelling errors and redundant phrases (e.g., participant re‐typed “that is” before providing their response).

Following pre‐processing, labels were transformed into sentence‐level semantic embeddings (*N* = 768 features) using a computational language processing model, BERT (Devlin et al., 2019), pre‐trained on uncased English text (https://github.com/hanxiao/bert-as-service). We used a hierarchical clustering algorithm to identify label embeddings that could not be clustered with other label embeddings for the same tile, given their distance in semantic space. These outlier labels (0.24%) were not used for social (or control) map generation.

Our goal was to objectively model the degree to which the content in a tile reflected a particular kind of semantically meaningful information (e.g., social information). Broadly speaking, to do this, we leveraged the idea that words that are lexically similar (i.e., occur in similar lexical contexts in natural language) are also conceptually similar (i.e., share common meaning) (Grand et al., 2022). Specifically, we assigned each tile a value (e.g., a social value) by comparing the sentence embedding for each tile to the average vector of sentence embeddings we knew contained a specific domain of semantic information, “landmarks” (e.g., social sentences) (Figure 2b). We constructed three landmarks in semantic space: social (primary analysis), object (control analysis), and place (control analysis). For each semantic domain, we collected 100 sentences from online sources that related to the semantic domain (see Table 2) and used BERT to obtain their average semantic embedding. For example, social landmark sentences included plot summaries from imdb.com, which contained descriptions of people, relationships, and emotional experiences; object landmark sentences included product descriptions for tools and electronic devices; and place landmark sentences included descriptions from hgtv.com of indoor and outdoor spaces. We then applied principal components analysis to these landmark embeddings to capture the most variability within them (Ringnér, 2008) and applied the derived coefficients to each of the label embeddings described above. To generate each domain‐specific semantic map (e.g., social), we first calculated the distance (cosine similarity) between each label embedding and the respective landmark embedding. We subtracted each distance from one to infer its domain similarity (e.g., how similar the label embedding for this tile is to the social landmark embedding). We normalized similarity values within each domain over all labels for all tiles and scenes and then averaged the scaled similarity scores of the labels provided for each tile. We plotted the average similarity value for each tile at its center coordinate in an equirectangular map and smoothed with a variable‐width Gaussian filter (John et al., 2019). To account for the tendency to scan the equator, we applied a multiplicative equator bias (Sitzmann et al., 2018), and finally, to better approximate the sparsity of fixations made by participants in each trial, we converted values below the 80th percentile to zero. We acknowledge that for many photospheres, this final thresholding step effectively distills the social map to a predictable set of regions that could have been identified by hand (e.g., faces, bodies). However, the benefit of our approach, even in these instances, is the objective assignment of a *unique* value at each location. For example, in a traditional area‐of‐interest (AOI) approach, two faces—one in the foreground and one more distant—would both be assigned the same binary value (i.e., 1); in contrast, our approach is sensitive to the relative importance of information from the same category (e.g., two faces) within a given scene (Figure 2c). All maps were normalized on a scale from 0.1 to 1. All in all, this procedure resulted in three “meaning maps” for each scene: social, object, and place (see Figure S6 for examples).

**TABLE 2:** Generation of semantic landmarks from web sentences

Landmark	Example sentences	Source
Social	*Faced with an unplanned pregnancy, an offbeat young woman makes an unusual decision regarding her unborn child*.	imdb.com
	*Two youngsters from rival gangs fall in love, but tensions between their respective friends build toward tragedy*.	
Object	*The Eufy RoboVac 11s is an affordable, frills‐free robot vacuum that's small enough to clean under even the lowest furniture*.	pcmag.com
	*There's a padded laptop compartment inside as well as plenty of places to slip in cards, pens, and other small things*.	
Place	*A cozy sleeping nook is flanked by two built‐in bookshelves to create a symmetrical look in this attic bedroom*.	hgtv.com
	*A widened hallway flows effortlessly between this bathroom and master bedroom thanks to a door‐free entryway*.	

### Materials—Stimulus presentation and VR display

Experimental stimuli were applied to a virtual environment created in Unity (www.unity3d.com), and the experimental routine was programmed using custom scripts written in C#. Stimuli were presented to participants via an immersive, headmounted VR display (Oculus Rift, Development Kit 2; low persistence OLED screen; 960 × 1080 resolution per eye; ~ 100° field of view; 75 Hz refresh rate). Participants stood wearing the head‐mounted VR display and headphones during the experiment. This setup offered participants a self‐directed opportunity to explore the naturalistic environment from an egocentric perspective, enabling them to actively explore each 360° environment naturally via eye‐movements and head turns.

### Materials—Eyetracking technical specs

Binocular, in‐headset eye‐trackers monitored gaze location (Pupil Labs: 120 Hz sampling frequency, 5.7 ms camera latency, 3.0 ms processing latency; 0.6 visual degrees accuracy, 0.08 visual degrees precision). Gaze location was recorded with custom scripts written in C# for Unity.

### Procedure—Data collection

Before putting on the VR headset, participants were given the following instructions about the experimental task: “You will visit 18 new places, and you will see each place three times. You can look all around, including behind you. Look around naturally, just as you would look around a new place in your day‐to‐day life.” Next, participants participated in three experimental phases: Practice, Calibration, and Experimental Trials.

During the practice phase, participants were introduced to VR to mitigate any potential effects of novelty or distraction due to inexperience on our main results. On each practice trial, participants explored two immersive scenes (static condition; 16 s each). Eyetracking confidence was monitored during the practice trials to ensure high‐quality pupil detection.

Second, during the calibration phase, eyetracking accuracy was validated using a 21‐point calibration routine. Prior to the start of each calibration routine, participants were encouraged to adjust the VR headset as needed for comfort. The calibration routine was repeated during the main experiment after every 10 scenes, as well as after any breaks in which the VR headset was removed, or when calibration drift from a pre‐trial fixation target was detected.

Third, during the experimental phase, 54 experimental trials (16 s each) were presented via the headmounted display: 18 scenes, each scene viewed in each of 3 conditions (static, dynamic, multisensory). We generated a randomized scene order for each participant, and we repeated the order three times so as to maximally separate scene presentations. Each scene was presented in a randomized condition order to minimize any confounding effects of presentation order on attention. The initial rotation angle for each scene was held constant across participants and conditions. After each experimental trial, participants returned to a virtual home screen (a platform surrounded by clouds), where they were instructed to take a break. To confirm accurate eyetracking before the next trial, participants were presented with a pre‐trial fixation target at screen center.

### Procedure—Data exclusion criteria

Experimental trials were excluded from data analysis if any of the following three criteria were met: (1) eye‐tracker confidence below 0.5 for more than 50% of the trial, (2) pre‐trial fixation deviation greater than 5° visual angle (DVA), or (3) participant scanned less than 50% of the photosphere (yaw). We included participants if they retained at least one third of experimental trials after applying trial‐level exclusion criteria.

### Procedure—Eyetracking data analysis

The continuous stream of raw *x* and *y* gaze coordinates collected via in‐headset eyetrackers was rectified with head position (pitch, yaw, roll), and transformed into latitude and longitude positions on a sphere (described in more detail in Haskins et al., 2020). Within each trial, a gaze point was labeled as invalid if: (1) it fell outside the field of view (i.e., greater than 50° from screen center in either the *x* and/or *y* direction), (2) pupil detection confidence was low (i.e., below 50%), or (3) no data was collected (e.g., during a blink).

### Procedure—Fixation definition

The continuous stream of gaze data collected via in‐headset eyetrackers was parsed following the procedure described in Haskins et al. (2020). To define fixations, we calculated the orthodromic distance and velocity between all consecutive time points in the gaze data. Time windows with mean absolute deviation (Voloh et al., 2019) of less than 50°/s were identified as potential fixations, and the fixation position was taken as the centroid of raw gaze points within the identified window. Fixations shorter than 100 ms, as well as the first fixation logged in each trial, were excluded.

### Procedure—Gaze map generation

**FIGURE 3:**
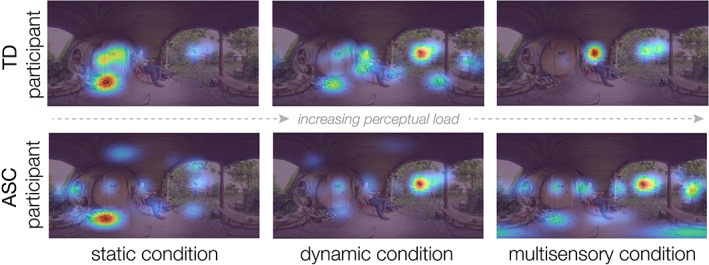
Gaze maps from two example participants. A typically developed (TD) participant (top) increasingly allocates their attention toward social information across conditions of increasing perceptual load. In contrast, an ASC participant (bottom) allocates attention to social information in each condition, but, relative to the TD participant, attention is less selectively correlated with social information under high load conditions.

Spatiotemporal gaze maps (e.g., see Figure 3) were generated for each participant and trial by plotting duration‐weighted fixations on an equirectangular map, smoothed with a variable‐width Gaussian filter to account for distortions of the equirectangular image at shorter latitudes (i.e., approaching the poles) (John et al., 2019). Gaze maps were then normalized for each trial on a scale from 0.1 to 1.

### Procedure—Quantifying social attention

Our primary dependent variable was the degree to which the distribution of a person's attention during a trial (i.e., gaze map) corresponded to the distribution of social information in each scene. Thus, we quantified participants' social attention by calculating the linear correlation between a participant's spatiotemporal gaze map and the social model described above. To account for distortions of the equirectangular image that might artificially inflate correlation values, we evenly sampled 10,000 locations on a sphere and calculated only the correlation between gaze and domain models at these indices (i.e., effectively sampling more densely at the center of the equirectangular images and less densely near the top/bottom). The same procedure was followed for control analyses, when quantifying participants' attention toward object and place information.

### Procedure—Quantifying entropy (control analysis)

To quantify gaze entropy, we generated a fixation density map for each trial and calculated a statistical measure of homogeneity of the resulting distribution of fixation coordinates (Açik et al., 2010). Here, fixations were not weighted by their duration; rather a value of 1 was plotted in each fixation location on an equirectangular map before applying a variable‐width filter.

## RESULTS

Our novel paradigm allowed us to measure participants' attentional selection in naturalistic, immersive scenes that were chosen to closely approximate the perceptual load of real‐world environments. We monitored participants' gaze as they actively explored a set of diverse, real‐world scenes (*N* = 18; Figure S1) in three experimental conditions that systematically modulated perceptual load (Figure 1). Each scene contained a rich variety of social information (e.g., individuals, interacting dyads), alongside object (e.g., cars, signs, electronics) and place (e.g., buildings, doors, walkways) information. For example, one scene depicted a woman in a snow‐covered landscape, surrounded by buildings, a tractor, and two children sledding nearby (Figure 2a). Participants were given no explicit task, but were encouraged to fully explore each scene, just as they would in daily life, by moving their eyes, head, and body.

We tested the hypothesis that differences in social attention (i.e., gaze directed to socially relevant scene information) between ASC and TD participants would be magnified by increasing perceptual load. Further, given that autism is a spectrum condition, we predicted that the degree of this interaction would scale with continuous measures of autistic traits across individuals. We quantified participants' social attention by measuring the degree to which their duration‐weighted fixation maps (henceforth “gaze maps”) corresponded to a continuous, computationally derived model of social information in each trial (Figures 2 and S6; see Section 2). To test whether real‐world perceptual load specifically impacts social attention, we also compared participants' gaze to two control models of nonsocial semantic information (object and place). Finally, we evaluated the impact of perceptual load on participants' low‐level gaze behavior (e.g., fixation number) to rule out the possibility that group differences emerge due to oculomotor, rather than attentional patterns.

We obtained high quality eyetracking data from nearly all participants (*N* = 1 ASC excluded for too few valid experimental trials; *N* = 1 ASC excluded from data analysis due to discomfort and frequent headset removal). Eyetracking calibration accuracy, measured via pretrial fixation target, was comparable between groups (TD mean deviation: 2.63 DVA, ASC mean deviation: 2.82 DVA, *t*(36) = 0.86, *p* > 0.05). Following trial‐level exclusions (see Section 2 for eyetracking data quality criteria), on average, 88% of trials from TD participants and 75% of trials from ASC participants were included in the analysis (*t*(36) = 2.76, *p* = 0.009). Importantly, groups were similar, on average, for low‐level fixation metrics underlying gaze maps for included trials (fixation duration, fixation number, summed fixation duration, all *p* > 0.05; Figure S2).

### Continuous, computationally derived social models predict gaze beyond spatial biases

Eyetracking studies commonly employ AOI analysis approaches to quantify how individuals deploy attention to scene content, but these techniques are limited. AOI techniques reduce scene content to binary dimensions (e.g., social vs. nonsocial) and often fail to capture contextual information. For example, two cell phones—one resting on a kitchen table and another being used in conversation—occupy different locations on a social–nonsocial continuum. Thus, we developed a novel method to better approximate the continuous distribution of socially relevant visual information present in real‐world scenes.

Here, we built upon an established approach for modeling domain‐general semantic information in a scene (Henderson & Hayes, 2017; Peacock et al., 2019), which has recently been applied to 360° environments (Haskins et al., 2020). Briefly, each scene was decomposed into smaller image tiles, and online participants provided contextually informed text descriptions of the content in each tile. We then combined these rich, contextually informed descriptions with state‐of‐the‐art computational language modeling (BERT) (Devlin et al., 2019) to approximate the distribution of real‐world social information. Critically, this approach enabled us to generate parallel control models of nonsocial scene information with the same verbal descriptions, thus enabling a direct comparison of our experimental manipulation with a single modeling process. Moreover, each model could be directly compared to the continuous distribution of individual participants' attention in that scene using an intuitive metric: correlation coefficient (Bylinskii et al., 2019).

We first confirmed that the social models for each scene corresponded with participants' attention, to a greater degree than a control, baseline model built to capture information‐neutral spatial viewing biases: the tendency to fixate along a scene's equator (Haskins et al., 2020; Sitzmann et al., 2018). Indeed, we find that the social maps for each scene are significantly correlated with participants' gaze behavior across trials in all three conditions (*t*(37) = 20.19, *p* < 0.001, CI:[0.34 0.42]). This correspondence holds when controlling for overlap between social models and the equator (*t*(37) = 18.94, *p* < 0.001, CI:[0.32 0.39]), demonstrating that the correspondence was not simply due to spatial viewing biases that might correlate with semantic content. Notably, the correlation between gaze and social maps was present for all participants, suggesting that all participants' gaze behavior was social information‐seeking to some degree.

### Reduced social attention in individuals with ASC emerges with perceptual load

Next, we aimed to evaluate the impact of real‐world perceptual load on social attention. Using a linear model, we conducted an analysis of variance (ANOVA) to evaluate the fixed effects of group (TD vs. ASC) and condition (static vs. dynamic vs. multisensory) on social attention. We included individual participants in the model as random effects, with the aim of increasing the generalizability of our findings beyond the specific participants included in this experiment (Bates et al., 2015).

**FIGURE 4:**
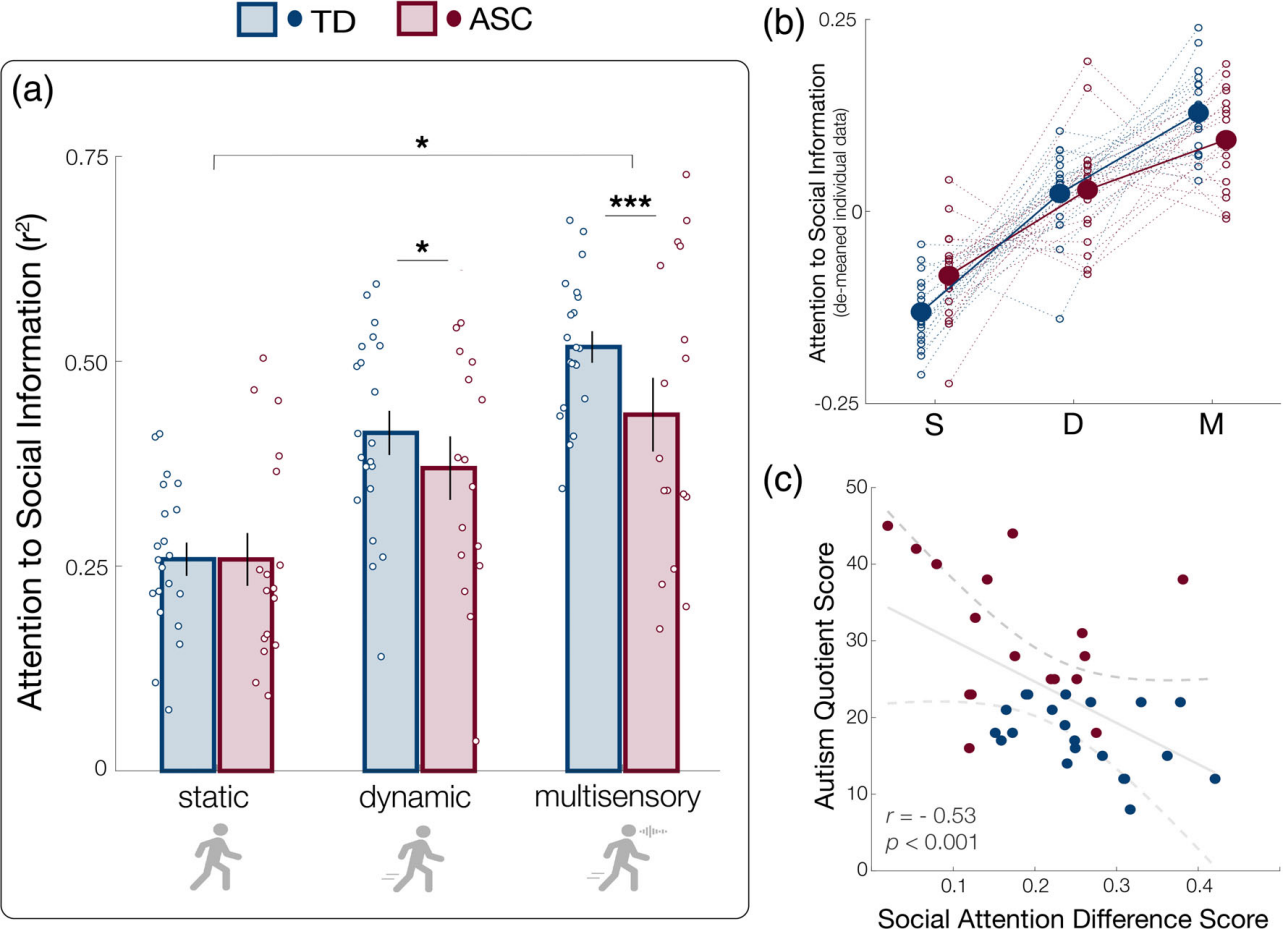
Group differences in social attention are modulated by perceptual load condition. (a) Social attention was quantified by measuring the degree to which participants' gaze corresponded to a continuous, computationally derived model of social information. We observed a significant interaction between perceptual load condition and group (*F*(2,1680) = 3.82, *p* = 0.022): ASC participants displayed reduced social attention relative to typically developed (TD) participants, only in higher perceptual load conditions (i.e., dynamic and multisensory, but not static). (b) Individual data from panel (a), de‐meaned to visualize the interaction between condition and group. For each gain in social attention that control participants receive across more complex, multisensory videos, the gain received by participants with autism was reduced. (c) Difference scores (“social vulnerability”) between individuals' social attention in high and low load conditions are significantly correlated with self‐reported autistic traits (*r* = −0.53, *p* < 0.001). In all plots, error bars represent 2 SEM. *p < 0.05; **p < 0.01; ***p < 0.001

Across all participants, social attention significantly increased across conditions (*F*(2,1680) = 29.50, *p* < 0.001; Figure 4a,b), demonstrating that as perceptual load increased, participants increasingly selectively attended to social information at the expense of other information sources. Importantly, we also observed a significant interaction between perceptual load condition and group (*F*(2,1680) = 3.82, *p* = 0.022; Figure 4a), where ASC participants displayed reduced social attention relative to TD participants, only in higher perceptual load conditions. Notably, this pattern of results does not change when including condition presentation order as a covariate in the model. Pairwise comparisons revealed significant group differences in dynamic and multisensory environments (dynamic: *p* = 0.04, *d* = 0.17; multisensory: *p* < 0.001, *d* = 0.43). In contrast, no group differences were observed in the static image condition (*p* > 0.05), demonstrating that group differences in social attention are not static, but emerge and are magnified in the presence of real‐world perceptual load.

### Perceptual load specifically impacts social attention

One possible explanation for the pattern of results we observe is that real‐world perceptual load impacts TD versus ASC participants' exploratory viewing behavior in a domain‐general way (i.e., not specific to social attention). For example, real‐world complexity may cause ASC participants to allocate attention less systematically, resulting in more distributed gaze patterns that are less correlated with localized, semantic information of any kind. We performed two control analyses to assess this possibility. First, to evaluate whether ASC participants' gaze became less systematic under higher perceptual load, we characterized gaze entropy, an established proxy of visual exploration (Açik et al., 2010), on each trial. As expected, gaze became less entropic (i.e., more systematic) with increasing perceptual load for both groups (*F*(2,1680) = 37.75, *p* < 0.001). However, we found no evidence of group differences in gaze entropy (*F*(1,1680) = 1.50, *p* = 0.22), and no evidence of an interaction between group and condition (*F*(2,1680) = 0.06, *p* = 0.95), suggesting that TD and ASC participants deployed their gaze in a manner that was comparably systematic (Figure S3).

Second, to test whether the pattern we observed reflects a specific impact of perceptual load on social attention, we performed the same analysis on two additional models of nonsocial semantic information for each scene: object and place information. These models were generated following the same approach employed for social models (Figure 2; see Section 2). Both of the control models significantly predict gaze overall (object model: *t*(37) = 23.86, *p* < 0.001, CI:[0.15 0.18]; place model: *t*(37) = 11.53, *p* < 0.001, CI:[0.05 0.08]), even when controlling for spatial viewing biases. We do not find evidence for the same condition modulation of group differences toward either object (*F*(2,1680) = 1.40, *p* = 0.25) or place information (*F*(2,1680) = 1.92, *p* = 0.15), demonstrating that the impact of perceptual load is not domain general, but specific to social attention (Figure S4).

### Perceptual load comparably impacts low‐level gaze behavior in both groups

We further confirmed that our results cannot be accounted for by low‐level group differences in gaze behavior, such as the number or duration of fixations used to construct participants' gaze maps. ANOVA analyses revealed a main effect of condition on both fixation duration and fixation number, such that in the static condition, participants made more frequent, shorter fixations, relative to the higher load conditions. Critically, we observed this effect in both groups, and found no group by condition interaction in either fixation number (*F*(2,1680) = 1.28, *p* = 0.28) or fixation duration (*F*(2,1680) = 0.83, *p* = 0.44), demonstrating that differences we observe in social attention are not accounted for by low‐level gaze metrics (Figure S5).

### Social vulnerability to perceptual load is correlated with autistic traits

We next characterized each individual participant's vulnerability to perceptual load by computing the difference between their average social attention in the highest load (multisensory) and lowest load (static) conditions. We tested the relationship between individuals' social vulnerability (i.e., difference scores) and self‐reported autistic traits using the autism‐spectrum quotient (Baron‐Cohen et al., 2001). Intriguingly, we find a significant negative correlation between social vulnerability and AQ scores across all participants (*r* = −0.53, *p* < 0.001), such that participants with the smallest changes in social attention across conditions reported the highest levels of autistic traits (Figure 4c). We observe this relationship in both groups separately, although it only reaches significance in the TD group (TD: *r* = −0.41, *p* = 0.032; ASC: *r* = −0.31, *p* = 0.11).

## DISCUSSION

The purpose of this study was to examine the causal relationship between real‐world perceptual load and reduced social attention in autism. We hypothesized that group differences in social attention would be magnified by the presence of dynamic and multisensory features of the broader environment—features that have often been excluded from traditional eyetracking paradigms. Our results support this hypothesis: we demonstrate reduced social attention is not an omnipresent group difference between individuals with autism and controls, but rather, one that emerges with increased perceptual load across experimental conditions. In control participants, social attention increased systematically with perceptual load, essentially doubling in the most complex, naturalistic condition. Importantly, however, social attentional gains in autism were smaller relative to control participants. In other words, for each “boost” in social attention that control participants receive in more complex, multisensory videos, the boost received by participants with autism was reduced. Across individuals in both groups, social vulnerability (i.e., the difference between social attention in high and low load conditions) was correlated with continuous measures of autistic traits. Critically, this vulnerability was specific to social information: we found no evidence of the same relationship between group differences and perceptual load, on attention toward either object or place information.

Social attention has been studied extensively in autism (Frazier et al., 2017), given its foundational importance in both typical and atypical social development (Klin et al., 2002). Here, we employed novel experimental and computational approaches to gain a new perspective on the nature of autistic group differences in social attention. The major methodological improvements are threefold. First, we employed a novel active viewing eyetracking paradigm, which allowed us to approximate the real‐world conditions where individuals actively engage, and where autistic individuals experience difficulty in daily life. Notably, a handful of previous studies have used mobile eyetracking to measure attention in immersive, real‐world environments with individuals with autism (Barzy et al., 2020; Noris et al., 2012; Yurkovic et al., 2021). However, VR offers key benefits over mobile applications, including experimental control over factors like timing and stimulus order, and the ability to immerse participants in a diverse set of complex environments. Moreover, recent work has demonstrated that an active viewing paradigm impacts nearly all aspects of gaze behavior and specifically increases viewers' attention to semantically meaningful information (Haskins et al., 2020), such as its social, nonsocial, and navigational affordances. Second, we created stimuli that allowed us to systematically vary perceptual features (i.e., dynamism, multisensory cues), while holding visual scene semantics constant. This allowed us to isolate the specific influence of perceptual load on social attention in autism. Third, we introduce a novel approach for modeling a continuous distribution of social information in real‐world scenes, which goes beyond a binary, subjectively defined AOI model (Hessels et al., 2016). This approach afforded a more nuanced characterization of participants' social attention by building into our models the rich, context‐informed social inferences that humans rapidly perform as they navigate real‐world environments.

These results represent a key step toward linking the eyetracking literature to the everyday experiences and difficulties of autistic individuals and advancing the clinical utility of eyetracking paradigms (Frazier et al., 2021; Murias et al., 2018). Specifically, our results speak to an ongoing puzzle in the social attention literature: group differences measured using eyetracking in laboratory studies are small (Chita‐Tegmark, 2016) and inconsistent (Guillon et al., 2014) relative to the day‐to‐day social difficulties that are hallmark of the condition and often readily apparent to trained clinicians. As a clear demonstration of this inconsistency, two recent meta‐analyses of the eyetracking literature have made contradictory conclusions regarding social attention in autism. In one review, the authors concluded that group differences in social attention were reliable and stable across age groups (Frazier et al., 2017). In a second review, the authors concluded essentially the opposite: differences in social gaze are not reliable and vary across development (Guillon et al., 2014). An existing account of the inconsistency in the literature on social attention in autism points to specific features of the social stimuli employed, such as speech and interactive social exchanges (Chawarska et al., 2012; Chevallier et al., 2015; Libertus et al., 2017; Macari et al., 2021; Shic et al., 2014). However, the focus of previous investigations has been on the impact of dynamic and multisensory features of social stimuli themselves (e.g., human facial movements) on social attention, rather than to the broader (nonsocial) environment. Here we propose that eyetracking studies may fail to capture real‐world group differences when experimental paradigms fail to capture naturalistic, complex features of real‐world viewing.

Our analysis primarily focused on social attentional differences, but it is also important to situate our results among previous demonstrations of domain‐general attentional processing differences in autism (Kaldy et al., 2011; Manning et al., 2015; Plaisted et al., 1998). Studies using simple visual tasks (e.g., searching for a target in a letter array) have shown that distractor stimuli are more readily processed when TD participants are attending under low perceptual load conditions (e.g., small array size, single feature target) than under high load conditions (e.g., large array size, conjunctive feature target), suggesting that selective attention is constrained by perceptual capacity limits (Lavie, 1995). Autistic individuals are differentially impacted by perceptual load in such lab‐based tasks: for example, in search paradigms, ASC participants continue to process distractor stimuli even under high perceptual load (Remington et al., 2009; Swettenham et al., 2014; Tillmann & Swettenham, 2017). Our results extend these findings to the social domain, and lend evidence to the notion of enhanced perceptual capacity in autism with a highly naturalistic paradigm. Across conditions of increasing perceptual load, control participants increasingly allocated their attention toward social information, largely ignoring other information sources as the perceptual demands increased. In contrast, despite demonstrating an overall selection priority for social information (i.e., main effect of condition), autistic participants' gaze was more widely distributed, even in higher load conditions. Though our present focus was on social *challenges* in autism, an important counter‐perspective to consider is *strength*‐based: specifically, our results suggest that in certain environments, autistic adults may be better than non‐autistic adults at maintaining a wide angle attentional focus despite increased perceptual demands. Of course, having enhanced perceptual capacity may contribute to the real‐world experience of sensory overload (Remington & Fairnie, 2017). A critical future application of virtual and augmented reality technologies will be to understand the particular tasks and contexts in which individuals' superior capacities can be used to their advantage for daily life.

Sensory traits are well‐described in the autism literature (Robertson & Baron‐Cohen, 2017), but their relationship to candidate diagnostic markers, like social attention, are poorly understood. Previous attempts to link sensory and social domains have relied heavily on evidence from self‐report measures: for example, among both neurotypical and autistic adults, higher levels of self‐reported autistic traits in the social domain (e.g., preferences for solitary activities; difficulty understanding jokes) are associated with higher levels of self‐reported abnormal sensory experiences (e.g., sensitivity to loud sounds or flickering lights) in both Western (Horder et al., 2014; Tavassoli et al., 2014) and Japanese cultures (Ujiie & Wakabayashi, 2015). Additionally, among children with autism, parent‐reported sensory traits correlate with both reported social difficulties and attention differences in naturalistic social scenes (Kojovic et al., 2019). Outside of self‐report measures, twin studies also suggest a link between these functional domains, as autistic sensory traits show relatively high heritability in twin studies, as well as high genetic overlap with social autistic traits (Taylor et al., 2018). Nonetheless, it remains unclear whether sensory traits causally influence real‐world social behavior. Our results provide rare evidence for a causal link between social and sensory processing in autism.

Our approach is not without limitations, and future work is needed to test both the generalizability and specificity of our results. Because our study was limited to a relatively small sample of adult participants with high cognitive profiles (i.e., average to above‐average nonverbal intelligence, verbal fluency), a key future direction will be to test that our results generalize across the phenotypic variability present in autistic individuals, as well as developmental timepoints. Furthermore, we acknowledge the set of perceptual features we have manipulated in this investigation in no way captures the breadth of perceptual experiences that autistic individuals and their families report as challenging. Thus, entire sensory domains that are beyond the scope of the current investigation (e.g., olfactory, tactile), may also critically influence individuals' social behavior in real‐world environments. In addition, we acknowledge certain limitations of our experimental setup. Our paradigm captures a key feature of naturalistic viewing: namely, that real‐world environments are visually immersive. However, real‐world environments might also be described as socially immersive. Viewers attend to social information, and in many cases, the social information (e.g., another person) is likewise attending to the viewer. The implication of this reciprocity in real‐world environments is that the task is not simply to socially attend but also to navigate potential social interactions. Previous work has demonstrated that the potential for social interaction alters social attention among neurotypical participants (Laidlaw et al., 2011). Our aim in this study was to bridge the gap between screen‐based studies of social attention and real‐world social difficulty in autism, but of course, investigations employing complementary methodologies (e.g., mobile eyetracking) are likely necessary to fully realize that goal. Relatedly, our stimuli were constrained by the need to hold semantic information spatially constant; thus, an important next step will be to employ more sensitive modeling approaches that enable us to test the generalizability of our results to more spatially entropic experimental stimuli that even more closely approximate daily experience (e.g., a person traversing through an environment). Finally, from a theoretical perspective, an important next step will be to understand the unique impact of perceptual load on social attention in autism. For example, we observed large group differences in social attention in the multisensory condition, which we attribute to an increase in perceptual load. Over longer temporal windows, however, linguistic cues are actively integrated with visual cues to guide attention and update the semantic meaning of scene content (e.g., “this tractor was given to me by my father”), which may also contribute to these results. An important future research direction will be to examine how the semantic meaning imparted by real‐time conversation influences attentional guidance and interacts with other load‐dependent cognitive capacities (e.g., working memory).

All in all, these results bring eyetracking studies of autism into a naturalistic, but controlled context. Within a complex, real‐world environment, our results indicate that reduced social attention may be an incomplete characterization of autistic social behavior. Instead, our results suggest that the key difference in autism is more nuanced and perhaps better captured by social vulnerability, particularly to the load of environmental complexity. These results mark a closer step toward understanding how individuals with autism navigate the rich, context‐informed social world of day‐to‐day life.

## AUTHOR CONTRIBUTIONS

Amanda J. Haskins and Caroline E. Robertson conceived the idea and designed the research. Amanda J. Haskins, Jeff Mentch, Thomas L. Botch, Brenda D. Garcia, and Alexandra L. Burrows collected the data. Amanda J. Haskins, Jeff Mentch, and Thomas L. Botch developed VR paradigm and eyetracking code. Amanda J. Haskins analyzed the data. Amanda J. Haskins and Caroline E. Robertson wrote the manuscript.

## CONFLICT OF INTEREST

The authors declare no conflicts of interest.

## Supporting information


**APPENDIX S1:** Supporting Information

## Data Availability

The data that support the findings of this study are available from the corresponding author upon reasonable request.
