# Supplementary material for: Reduced social attention in autism is magnified by perceptual load in naturalistic environments
Source: Autism Res. 2022 Oct 7;15(12):2310–23. doi: 10.1002/aur.2829 (PMC10092155; doi:10.1002/aur.2829)
Supplement: Supplementary file 1 — APPENDIX S1: Supporting Information [file AUR-15-2310-s001.docx]

**Supplemental Information**

**Reduced social attention in autism is magnified by perceptual load in naturalistic environments**

Amanda J. Haskins^1^, Jeff Mentch^2,3^, Thomas L. Botch^1^, Brenda D. Garcia^1^, Alexandra L. Burrows^1^, Caroline E. Robertson^1^

^1^ Department of Psychological & Brain Sciences, Dartmouth College, Hanover, NH, 03755, USA.

^2^ Speech and Hearing Bioscience and Technology, Harvard University, Boston, MA, 02115, USA.

^3^ McGovern Institute for Brain Research, MIT, Cambridge, MA, 02139, USA.

*Correspondence concerning this article should be addressed to: ajh.gr@dartmouth.edu (A.J.H.), cerw@dartmouth.edu (C.E.R.)

| *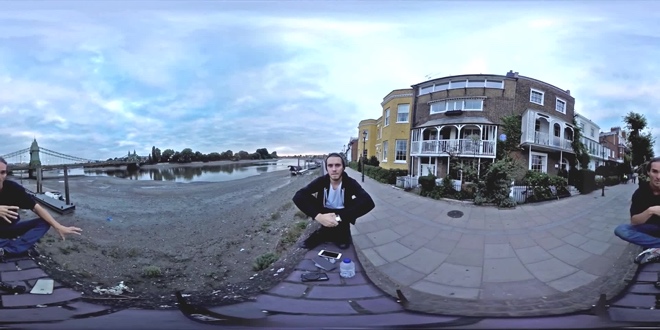* | *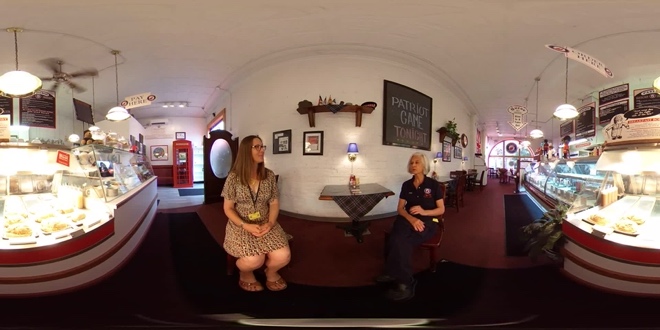* | *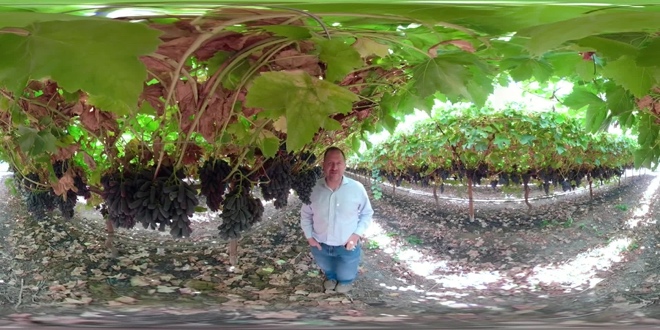* |
| --- | --- | --- |
| [*Videosphere source link*](https://www.youtube.com/watch?v=eTlFhufxzhk) | [*Videosphere source link*](https://www.youtube.com/watch?v=cfuNF1ac8hg) | [*Videosphere source link*](https://www.youtube.com/watch?v=9EKugQMdtPY&list=PLnCKvJ43E7oKDrMgaCPRN7Ql6hul2x1-T&index=51) |
| *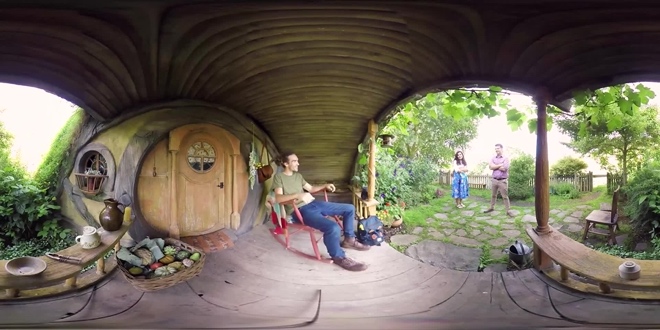* | *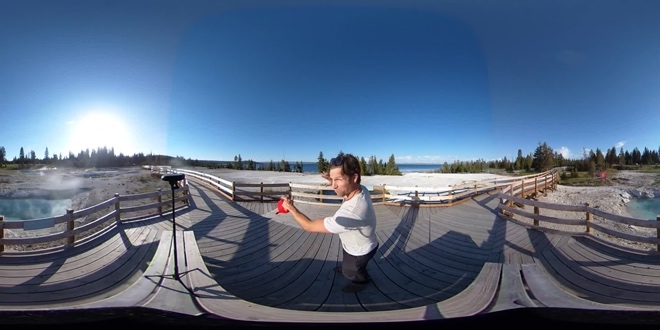* | *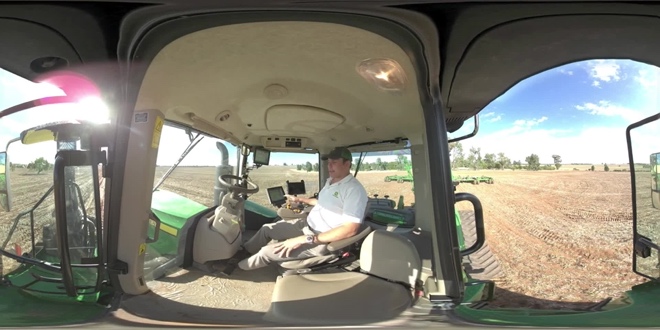* |
| [*Videosphere source link*](https://www.youtube.com/watch?v=7SerosZOvvQ) | [*Videosphere source link*](https://www.youtube.com/watch?v=QAhKxatumpM&list=PLvgu5VYoutJ-4uejqqhotTx0WGET8wtho&index=5) | [*Videosphere source link*](https://www.youtube.com/watch?v=8-nSmAJar6k&list=PLbI51UxWCtCLC46E54osKP8or5XzoYP4D&index=7) |
| *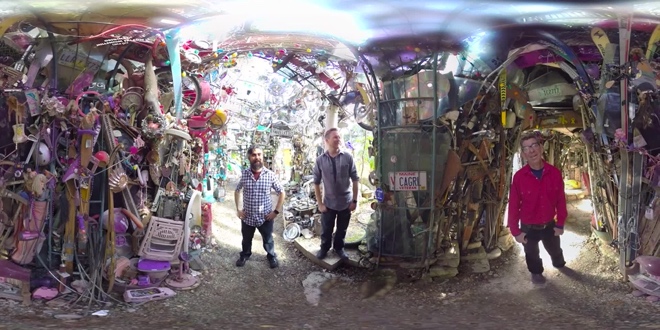* | *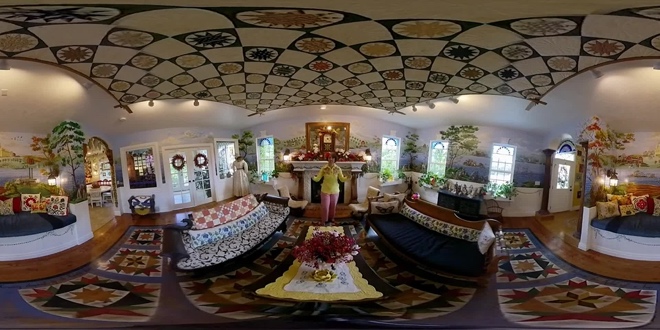* | *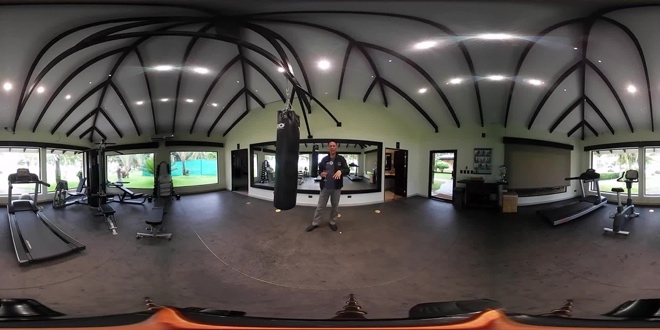* |
| [*Videosphere source link*](https://www.youtube.com/watch?v=-GREoE1qcyI) | [*Videosphere source link*](https://www.youtube.com/watch?v=bLrL_cmtet0) | [*Videosphere source link*](https://www.youtube.com/watch?v=dgISoqfQrsI) |
| **Figure S1: Full set of real-world scenes shown to participants.** Each scene (N = 18 total; 16s each) was presented in three experimental conditions: static photosphere (as shown here), dynamic videosphere (muted video), and multisensory videosphere (original source links provided). All scenes depicted rich, engaging information of both social and nonsocial quality, as determined by pilot participants’ group-level attention maps. | | |
| *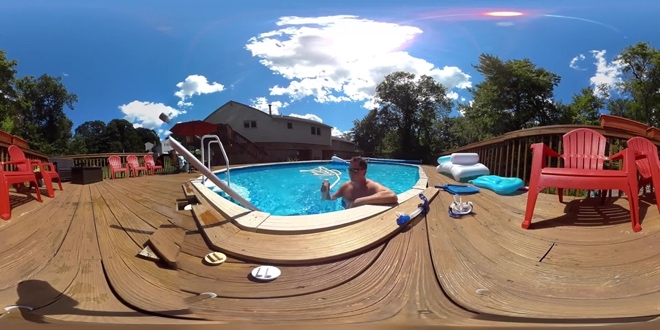* | *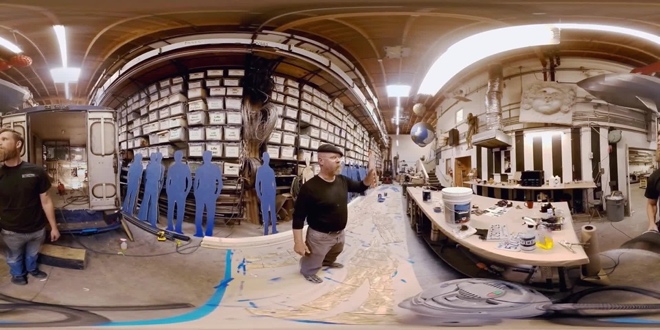* | *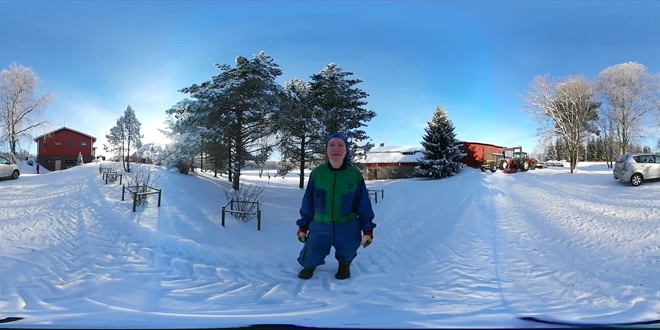* |
| [*Videosphere source link*](https://www.youtube.com/watch?v=jqYQ_Lx9wLE) | [*Videosphere source link*](https://www.youtube.com/watch?v=jBCs10yczfY) | [*Videosphere source link*](https://www.youtube.com/watch?v=q0iA1OhRprY) |
| *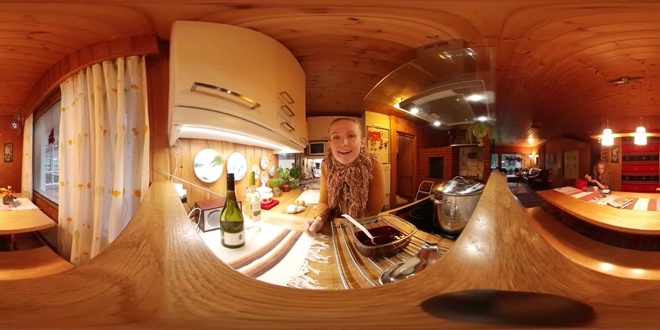* | *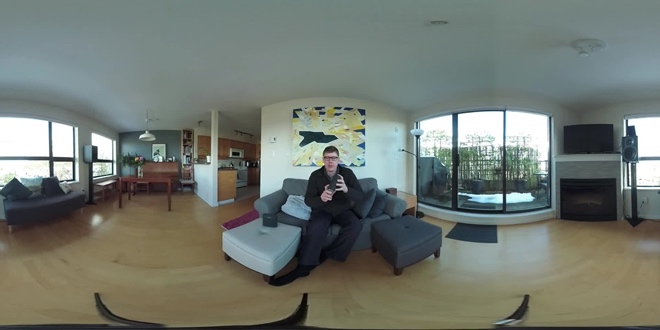* | *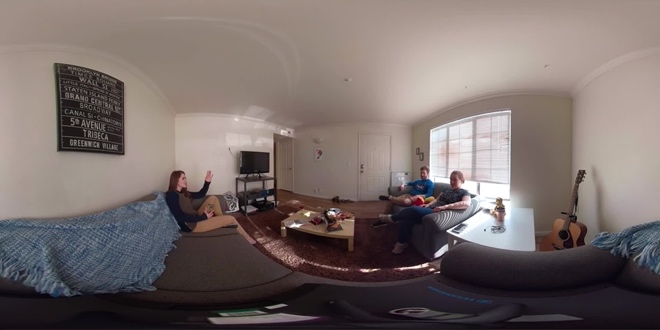* |
| [*Videosphere source link*](https://www.youtube.com/watch?v=xRk1Hj7yq0A&list=PLG8t0GaNncUl1lVNF-kP3Z2q7o0tpZ0WI&index=56) | [*Videosphere source link*](https://www.youtube.com/watch?v=nkyK2w0UmkM) | [*Videosphere source link*](https://www.youtube.com/watch?v=CqdRG_JBD5U) |
| *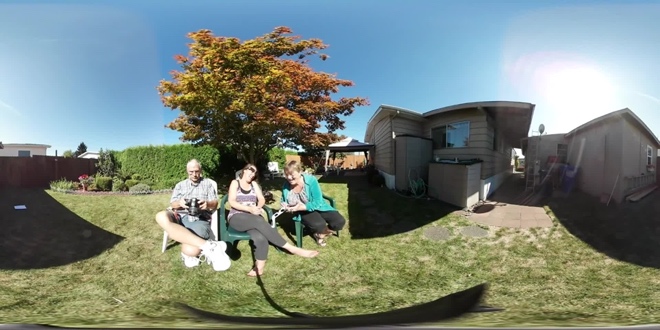* | *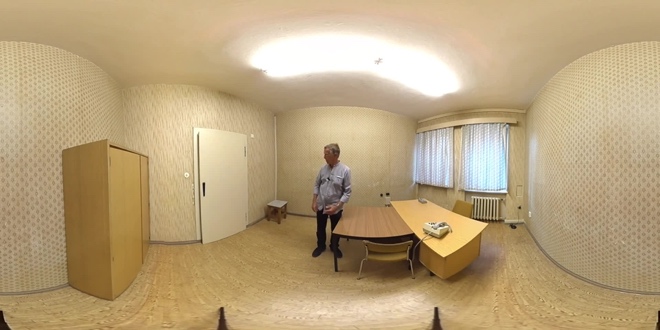* | *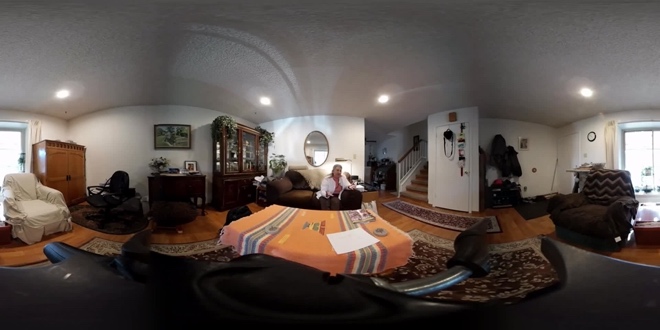* |
| [*Videosphere source link*](https://www.youtube.com/watch?v=b7e_fGuLD3w) | [*Videosphere source link*](https://www.youtube.com/watch?v=4vt8Qx4L_VE) | [*Videosphere source link*](https://www.youtube.com/watch?v=vDOyNQKVkvg) |
| **Figure S1 (continued): Full set of real-world scenes shown to participants.** Each scene (N = 18 total; 16s each) was presented in three experimental conditions: static photosphere (as shown here), dynamic videosphere (muted video), and multisensory videosphere (original source links provided). All scenes depicted rich, engaging information of both social and nonsocial quality, as determined by pilot participants’ group-level attention maps. | | |

| **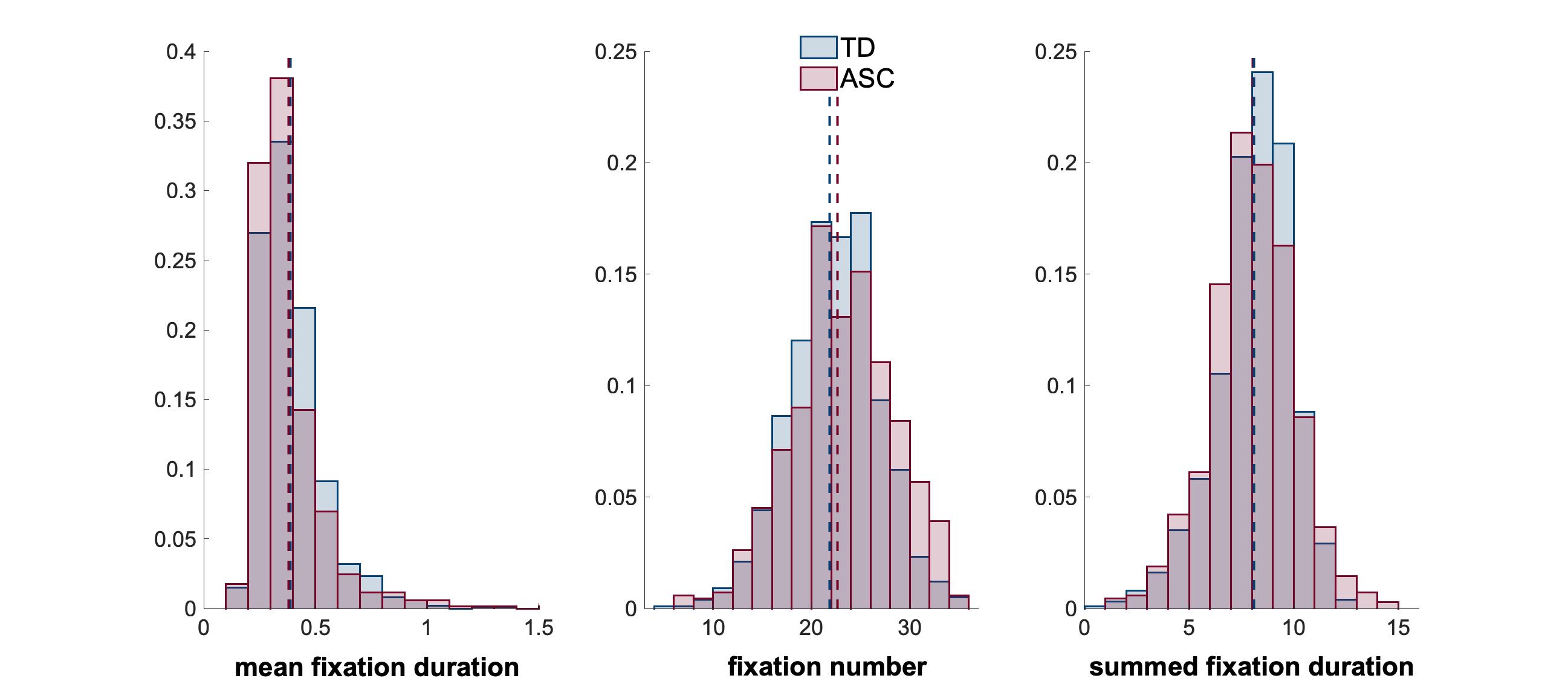**  Proportion of trials |
| --- |
| **Figure S2: Characteristics of fixations underlying gaze maps are comparable between groups.** We observed comparable low-level gaze characteristics among TD and ASC participants. Across conditions, mean fixation duration (left), fixation number (center), and total time in fixation (right) per trial were comparable for both groups (all group comparisons *p* > 0.05). Dotted lines depict group means. |

| 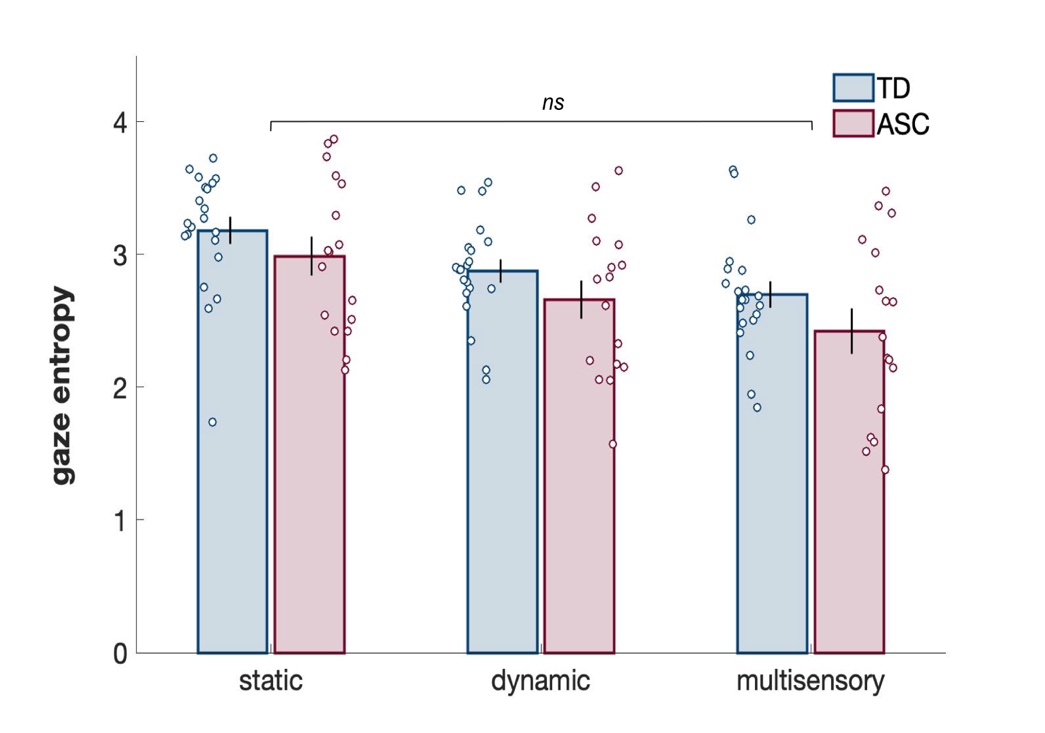 |
| --- |
| **Figure S3: Perceptual load comparably impacts gaze entropy in both groups.** To evaluate the possibility that perceptual load caused autistic participants’ visual exploration to be less systematic, we calculated the gaze entropy for each trial. We observed a significant main effect of condition on gaze entropy (*F*(2,1680) = 37.75, *p* < 0.001), such that gaze is least entropic (i.e., most systematic) in high load conditions. We found no evidence of group differences in gaze entropy (*F*(1,1680) = 1.50, *p* > 0.05), and no evidence of an interaction between group and condition (*F*(2,1680) = 0.06, *p* > 0.05) |

| 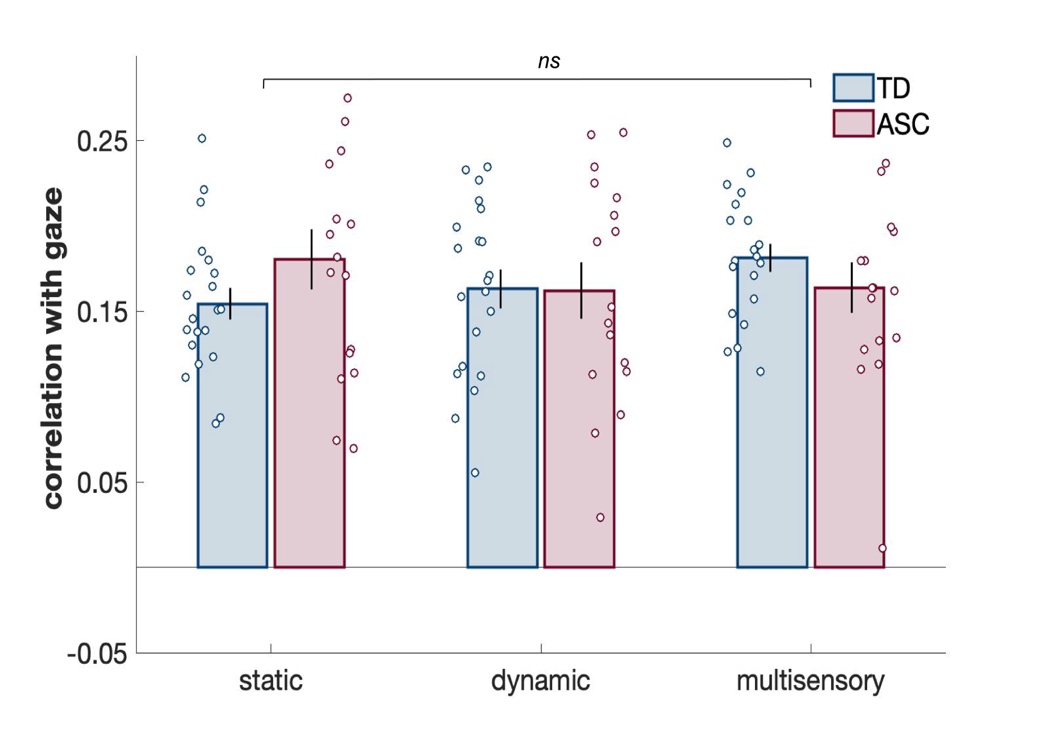  **object** | 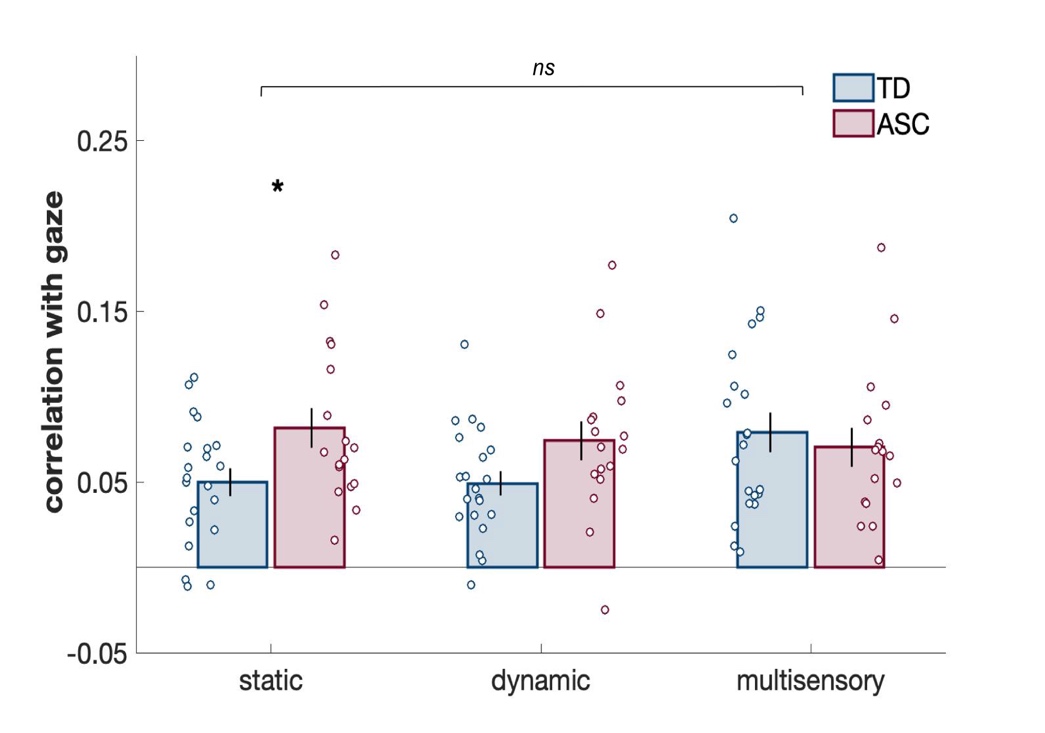  **place** |
| --- | --- |
| **Figure S4: Perceptual load does not impact attention to object or place information.** To test whether the impact of perceptual load was specific to social attention, we compared participants’ gaze to two additional, nonsocial models: object and place. We do not find evidence for the same condition modulation of group differences toward either object (*F*(2,1680) = 1.40, *p* > 0.05) or place information (*F*(2,1680) = 1.92, *p* > 0.05). | |

| 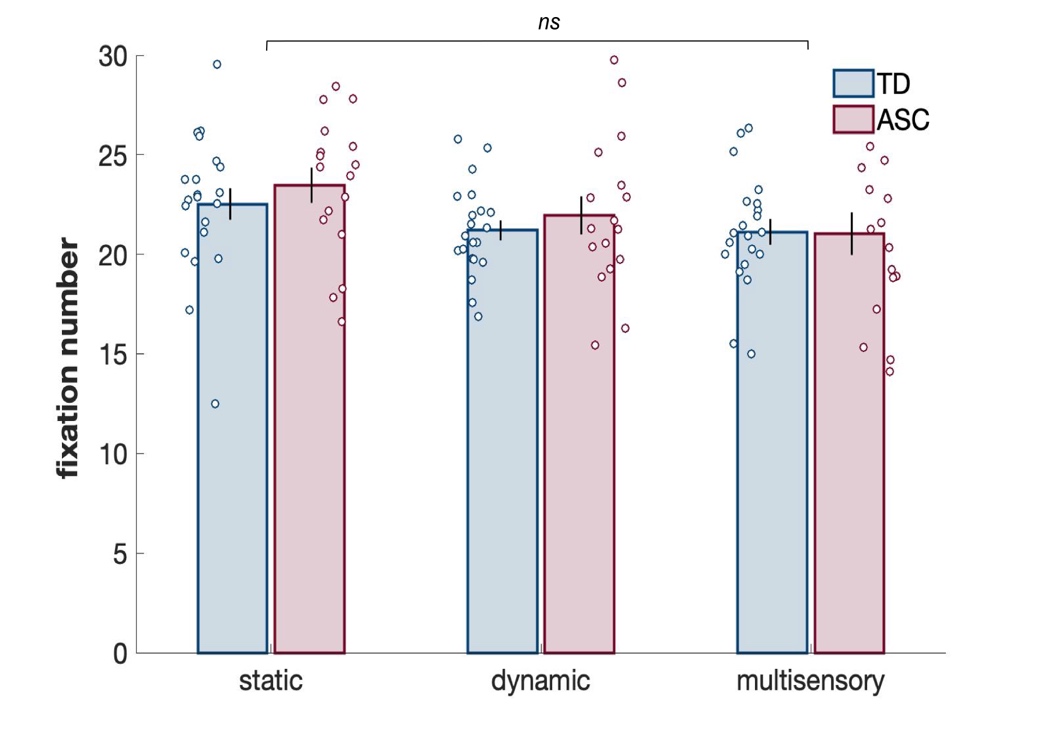 | 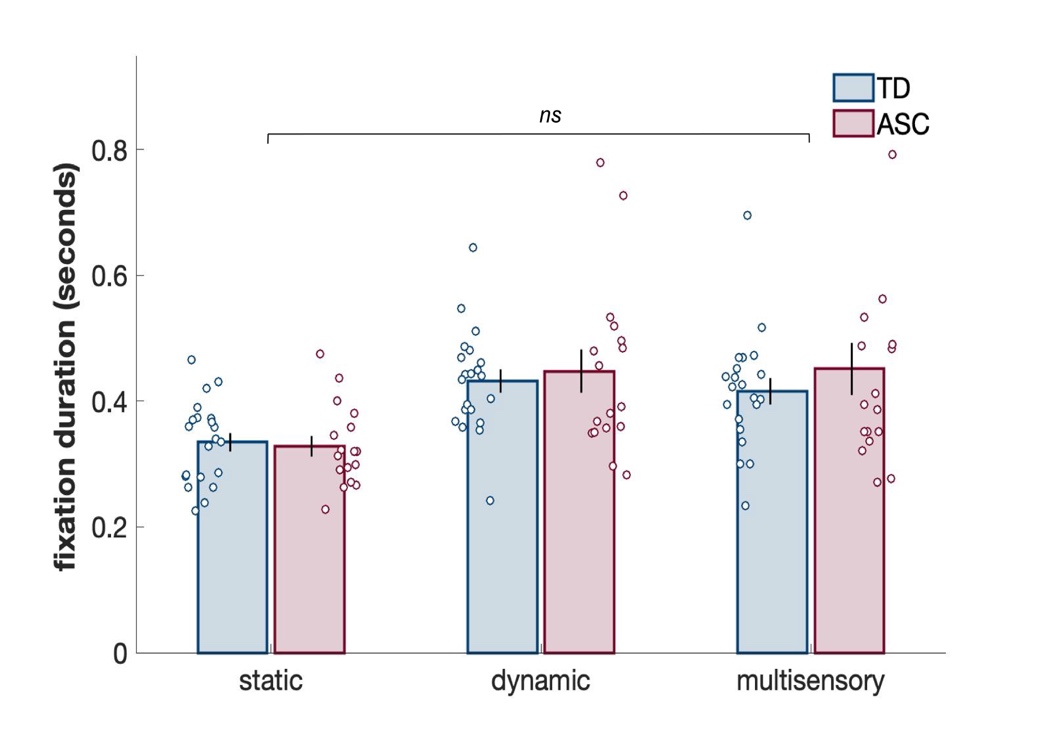 |
| --- | --- |
| **Figure S5. Perceptual load comparably impacts low-level gaze behavior in both groups.** Participants in both groups made more frequent, shorter fixations in the static photosphere condition, relative to the higher load conditions. However, we found no evidence for a group by condition interaction in either fixation number (*F*(2,1680) = 1.28, *p* > 0.05) or fixation duration (*F*(2,1680) = 0.83, *p* > 0.05). | |

| 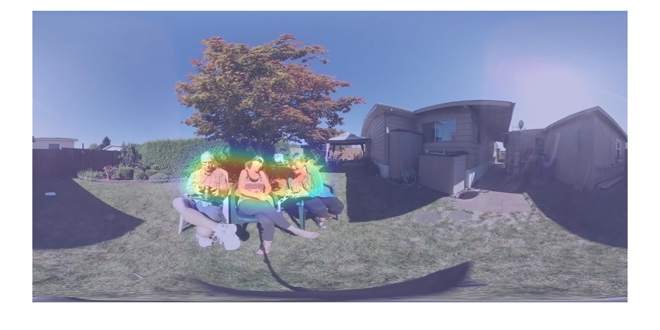 | 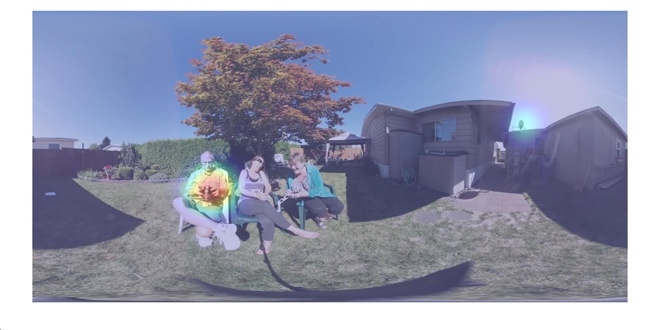 | 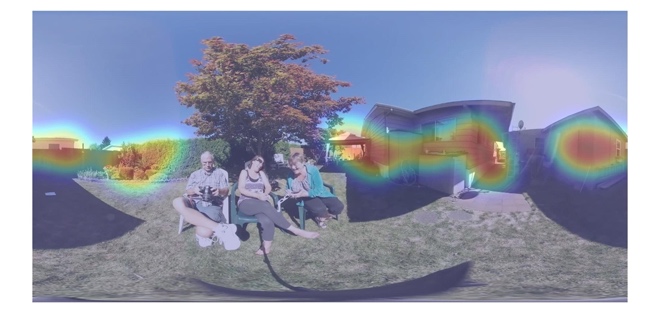 |
| --- | --- | --- |
| 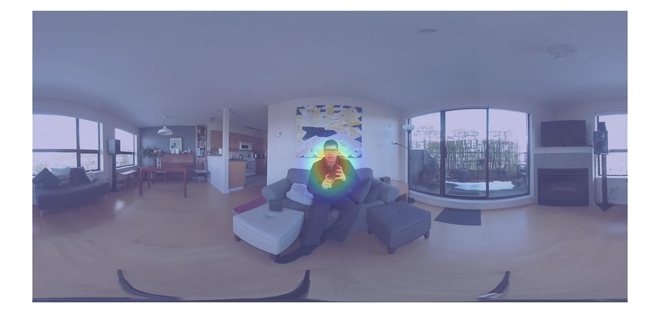 | 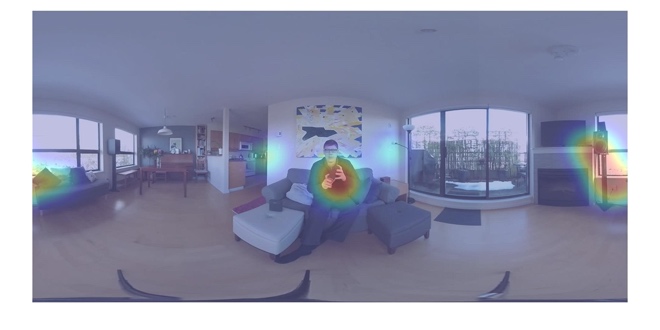 | 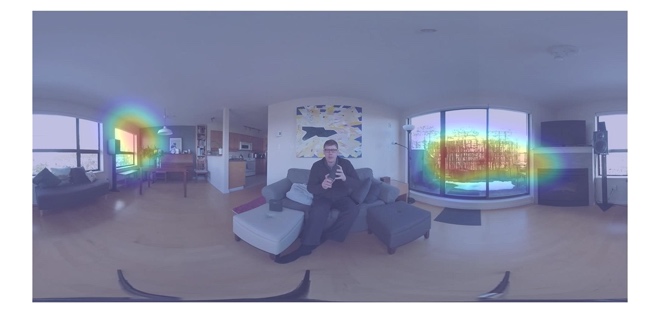 |
| **Figure S6: Example social, object, and place models.** For each scene (N = 18), we combined online participants’ verbal descriptions with computational language modeling to generate three continuous models of semantic information. Left: Social models (primary analysis) were generated by comparing verbal descriptions to a “social landmark” (i.e., the average embedding in the semantic space of a natural language model (BERT) for sentences about people, relationships, and emotional experiences). Scene regions of high social value included faces, bodies, and items being interacted with (i.e., looked at, touched). Middle: Object models (control analysis) were generated by comparing verbal descriptions to an “object landmark’ (i.e., the average sentence embedding for sentences about tools, gadgets, and other manipulable items). Scene regions of high value in the object model included cameras, phones, a TV satellite, and audio speakers. Right: Place models (control analysis) were generated by comparing verbal descriptions to a “place landmark” (i.e., the average sentence embedding for sentences describing indoor and outdoor places). Scene regions of high place value included fences, walkway, windows, and doors. See methods for more details. | | |
